# Micro-Bypass Implantation for Primary Open-Angle Glaucoma Combined with Phacoemulsification: 4-Year Follow-Up

**DOI:** 10.1155/2015/795357

**Published:** 2015-10-26

**Authors:** Antonio Maria Fea, Giulia Consolandi, Marta Zola, Giulia Pignata, Paola Cannizzo, Carlo Lavia, Teresa Rolle, Federico Maria Grignolo

**Affiliations:** Ophthalmic Clinic, Department of Surgical Sciences, University of Turin, 10122 Turin, Italy

## Abstract

*Purpose*. To report the long-term follow-up results in patients with cataract and primary open-angle glaucoma (POAG) randomly assigned to cataract surgery combined with micro-bypass stent implantation or phacoemulsification alone. *Methods*. 36 subjects with cataract and POAG were randomized in a 1 : 2 ratio to either iStent implantation and cataract surgery (combined group) or cataract surgery alone (control group). 24 subjects agreed to be evaluated again 48 months after surgery. Patients returned one month later for unmedicated washout assessment. *Results*. At the long-term follow-up visit we reported a mean IOP of 15,9 ± 2,3 mmHg in the iStent group and 17 ± 2,5 mmHg in the control group (*p* = NS). After washout, a 14,2% between group difference in favour of the combined group was statistically significant (*p* = 0,02) for mean IOP reduction. A significant reduction in the mean number of medications was observed in both groups compared to baseline values (*p* = 0,005 in the combined group and *p* = 0,01 in the control group). *Conclusion*. Patients in the combined group maintained low IOP levels after long-term follow-up. Cataract surgery alone showed a loss of efficacy in controlling IOP over time. Both treatments reduced the number of ocular hypotensive medications prescribed. This trial is registered with: NCT00847158.

## 1. Introduction

Primary open-angle glaucoma (POAG) accounts for at least 90% of all glaucoma cases and affects primarily 3 million people in the United States. Cedrone et al. reported a 3,2% incidence of POAG in the population-based Ponza eye study [1]. Increased intraocular pressure (IOP) in POAG is due to progressive obstruction of drainage which may lead to the damage of the optic nerve and subsequently blindness, if left untreated. Moreover, glaucoma and cataract incidence increase with age and are frequently found in the same patient [2, 3]. Thus, cataract surgery may be a good management option in medically controlled nonsevere glaucoma with a mild reduction in IOP [4, 5].

The iStent (Glaukos Corporation, Laguna Hills, CA) was developed to bypass the trabecular meshwork and create a direct route from the anterior chamber to Schlemm's canal. This ab interno device is implanted using the same temporal clear corneal incision used for cataract surgery. The iStent has been shown to lower IOP while reducing ocular hypotensive medication usage in prior studies involving eyes randomly assigned to cataract surgery and iStent implantation or cataract surgery alone with follow-up through 1 year to 24 months [6–9]. We reported in a previous study our findings involving subjects with POAG randomly assigned to phacoemulsification with micro-bypass stent implantation or phacoemulsification alone with a follow-up of 16 months [6]. IOP was statistically significantly lower in the combined group both before and after washout as compared to the control group. In addition, the mean number of medications was significantly lower in the combined group versus the control group. The purpose of this recent work was to report on long-term results through a minimum of 4 years on the original cohort of subjects.

## 2. Materials and Methods

### 2.1. Subjects and iStent

This prospective randomized study comprised 36 subjects with cataract and POAG. Subjects were required to have an IOP > 18 mmHg in three different visits while using at least one ocular hypotensive medication and a corrected distance visual acuity worse than 20/80. Key exclusion criteria were glaucoma other than POAG, cloudy cornea, potentially inhibiting gonioscopic view of the angle, peripheral anterior synechiae, prior ocular surgery, diabetic retinopathy, and age-related macular degeneration. Subjects were randomized in a 1 : 2 ratio to either iStent implantation in conjunction with cataract surgery (combined group) or cataract surgery alone (control group). The micro-bypass iStent (Glaukos Corporation, Laguna Hills, CA) is a heparin-coated L-shaped device (Duraflo, Edwards Lifesciences, Irvine, CA) made from titanium with a length of 1.0 mm and height of 0.33 mm and snorkel with a diameter of approximately 120 *μ*m. When implanted, the snorkel is located in the anterior chamber and the open half-pipe lumen (foot) is located in Schlemm's canal. This ab interno device establishes a patent bypass through the trabecular meshwork to Schlemm's canal to restore continuous physiologic outflow.

### 2.2. Surgical Technique

All subjects underwent standard clear corneal phacoemulsification with IOL implantation [6]. All preoperative peribulbar anesthesia dosing was considered current standard of care. Subjects implanted with the iStent (combined group) underwent stent implantation after IOL implantation. The stent was guided into the canal of Schlemm by ab interno gonioscopy using a Swan-Jacobs gonioscope. If no complications occurred during phacoemulsification, acetylcholine was injected into the anterior chamber after IOL implantation to constrict the pupil. The anterior chamber was then filled with an ophthalmic viscosurgical device to reform the anterior chamber and provide more clearance in the angle. With the stent on the tip of the applicator, the anterior chamber was traversed with the applicator and the trabecular meshwork located. The leading edge of the stent was gently slid through the trabecular meshwork and into the canal of Schlemm at the nasal position (3 to 4 o'clock in right eye; 9 to 8 o'clock in left eye) with the tip of the stent directed inferiorly. If there was difficulty with insertion at the primary location, a location of approximately 0.5 o'clock inferiorly was attempted and the surgeon continued to move inferiorly as needed for subsequent attempts. Next, the stent was released by pushing the button on the applicator. After the position of the stent was verified, the applicator was withdrawn.

### 2.3. Postoperative Follow-Up

Following the postoperative course described in the original paper, subjects were contacted again after approximately 4 years to determine their availability and willingness to undergo participation in a follow-up evaluation [6]. A total of 24 subjects agreed to participate. Subjects underwent an initial long-term evaluation, at which time they were instructed to discontinue ocular hypotensive medication and return 1 month later for an unmedicated assessment (washout evaluation).

### 2.4. Statistical Analysis

The efficacy analysis comprises all available long-term data on medicated IOP, ocular hypotensive medication use, and postmedication washout IOP. For continuous variables, 2-sample *t*-tests were used to assess between-group differences. Fisher exact tests were used to compare categorical outcomes between groups. An *α* level of 0,05 was considered statistically significant. All statistical tests were performed using PC-SAS (Version 9.1.3).

## 3. Results and Discussion

### 3.1. Patients Demographics

**Figure 1:**
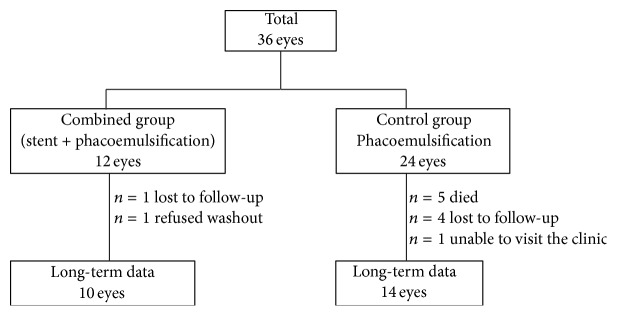
Subject accountability: a total of 10 eyes in the combined group and 14 eyes in the control group had long-term follow-up data.

Of the original cohort of 36 randomized subjects, 24 subjects were available for long-term follow-up assessment. A total of 5 subjects died, 5 subjects were lost to follow-up, one subject refused washout, and one subject could not travel to the clinic (Figure 1).

### 3.2. Intraocular Pressure

At baseline, mean IOP was 17,8 ± 2,7 mmHg in the combined group and 16,7 ± 3 mmHg in the control group (Table 1).

**Figure 2:**
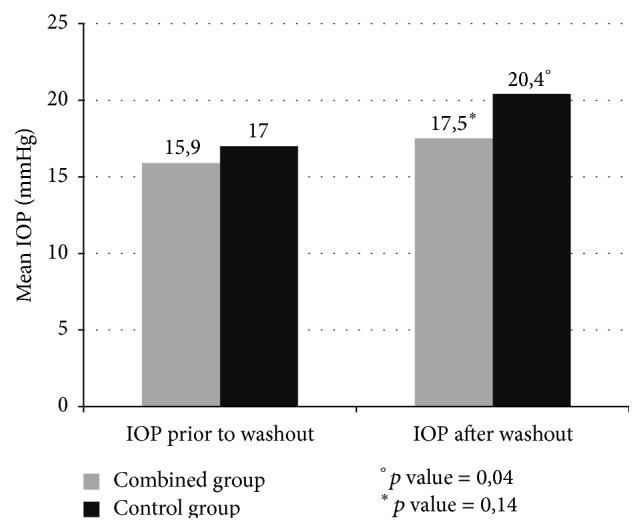
Mean intraocular pressure (IOP) at long-term follow-up before and after washout of ocular hypotensive medications at long-term follow-up (*p* values prior to and after washout: 0,14 in the combined group and 0,04 in the control group).

This difference was not statistically significant (*p* = NS). At the month 12 visit after washout, mean IOP was 16,1 ± 2 mmHg and 18,4 ± 3,1 mmHg in the iStent and the control group, respectively, a difference that was statistically significant (*p* = 0,05). Long-term follow-up visit before washout reported a mean IOP of 15,9 ± 2,3 mmHg in the iStent group and 17 ± 2,5 mmHg in the control group (*p* = NS). After the washout the IOP was 17,5 ± 2,3 mmHg in the combined group versus 20,4 ± 3,2 mmHg in the control group. In the combined group we found no statistically significant difference between before and after washout (*p* = 0,14) while it was found in the control group (*p* = 0.04). At the long-term follow-up visit after washout we found a difference of 14,2% between groups for mean IOP reduction (*p* = 0,02) (Figure 2). The difference between baseline and after washout IOP was 0,3 mmHg in the combined group versus 3,7 mmHg in the control group.

### 3.3. Ocular Hypotensive Medications

At baseline, the mean number of ocular hypotensive medications used was 1,9 ± 0,9 in the combined group and 1,8 ± 0,7 in the control group, a difference that was not statistically significant (*p* = NS). At 12 months, the mean number of medications was 0,4 ± 0,7 in the treatment group and 1 ± 1 in the control group (Table 2).

These reductions were statistically significant compared to baseline values (*p* = 0,003 for treatment group and *p* = 0,01 for the control group).

At long-term follow-up patients in the combined group used 0,5 ± 0,8 medications, a difference statistically significant compared to baseline (*p* = 0,005). Patients in the control group also had a significant reduction in the mean number of ocular hypotensive medications used (0,9 ± 1 medications; *p* = 0,01). No statistically significant difference was reported between groups at any visit.

### 3.4. Safety

The majority of patients had an improvement of UDVA and CDVA after phacoemulsification and intraocular lens (IOL) implantation. No significant differences were reported between groups at 12- and 48-month follow-up. No postoperative stent-related adverse events were observed in these eyes through 48 months. IOP was well controlled in both groups throughout the entire follow-up period; no secondary surgical intervention was required to control IOP.

In this study, the implantation of the iStent combined with cataract surgery resulted in a greater IOP reduction at long-term follow-up compared to cataract surgery alone. Ocular hypotensive medications used were reduced, both in the combined and in the control group. The micro-bypass stent combined with phacoemulsification constantly reduced IOP throughout the entire study, starting from 17,8 ± 2,7 mmHg at baseline to 16,1 ± 2 mmHg at 12 months and finally to 15,9 ± 2,3 mmHg at 48 months. Mean IOP in patients with cataract and POAG treated with phacoemulsification alone, increased at follow-up visits after washout. At long-term follow-up after washout, IOP in the control group was significantly greater than at baseline (20,4 ± 3,2 mmHg versus 16,7 ± 3 mmHg, *p* = 0,002) and a 14,2% difference compared to the combined group was reported, which was statistically significant (17,5 ± 2,3 mmHg in the combined group versus 20,4 ± 3,2 mmHg in the control group, *p* = 0,02). The results show how the iStent implantation combined with cataract surgery maintains its efficacy in lowering IOP in the long term, as reported in previous studies [6–9]. Mean IOP in the control group tended to rise at 48-month follow-up in our study. This data suggests a loss of efficacy with time for phacoemulsification alone, as previously reported in other studies [6–10]. The decrease in the number of glaucoma medications in our study is similar in both groups (1,4 ± 0,8 the difference observed in the combined group versus 0,9 ± 1 in the control group). A similar reduction is observed in other studies [6, 8, 10]. The reduction in the number of medications for chronic use in POAG remains a fundamental question with a view to improve patients compliance and to reduce conjunctival inflammation, preserve patients ocular surface integrity, and prevent from reduction in the success rate of subsequent trabeculectomy [11, 12]. With regard to safety, no adverse events related to the stent implantation were observed.

## 4. Conclusions

There are a number of strengths in this study. The length of its follow-up is certainly one of them, being one of the longest to be reported in literature to our knowledge [10]. Moreover, the patients' cohort included in the study presented with an increasingly common situation—primary open-angle glaucoma—encountered concomitantly with cataract. Our results are a measure of effectiveness more than a measure of efficacy.

Our study is not without limitations. After the first 12 months patients were referred back to their ophthalmologists, so IOP lowering medications were not prescribed based on a standardized protocol. This bias might be a reason for the lack of statistical significance in the mean number of medications. This is also why the results obtained after washout should be considered more significant. Finally, the number of patients evaluated at follow-up was small, with 10 eyes in the treatment group and 14 eyes in the control group. Our results need confirmation in larger randomized controlled clinical trials.

In conclusion, patients having a combined cataract surgery with iStent implantation maintained low IOP levels after 48 months of follow-up. Cataract surgery alone showed a loss of efficacy in controlling IOP over time. Both treatments reduced the number of ocular hypotensive medications prescribed.

## Figures and Tables

**Table 1:** Mean IOP values (mmHg) by visit.

	Baseline	12 months	12 months wo.	48 months	48 months wo.
Treatment group (SD)	17,8 (2,7)	14,7 (1,3)	16,1 (2)	15,9 (2,3)	17,5 (2,3)
Control group (SD)	16,7 (3)	15,6 (1,1)	18,4 (3,1)	17 (2,5)	20,4 (3,2)

SD: standard deviation; wo.: washout.

**Table 2:** Ocular hypotensive medications by visit (mean values).

	Baseline	12 months	48 months
Treatment group (SD)	1,9 (0,9)	0,4 (0,7)	0,5 (0,8)
Control group (SD)	1,8 (0,7)	1 (1)	0,9 (1)

SD: standard deviation.

## References

[1] Cedrone C., Mancino R., Ricci F., Cerulli A., Culasso F., Nucci C. (2012). The 12-year incidence of glaucoma and glaucoma-related visual field loss in Italy: the Ponza eye study. *Journal of Glaucoma*.

[2] Lau J. T. F., Lee V., Fan D., Lau M., Michon J. (2002). Knowledge about cataract, glaucoma, and age related macular degeneration in the Hong Kong Chinese population. *British Journal of Ophthalmology*.

[3] Friedman D. S., Jampel H. D., Lubomski L. H. (2002). Surgical strategies for coexisting glaucoma and cataract: an evidence-based update. *Ophthalmology*.

[4] Poley B. J., Lindstrom R. L., Samuelson T. W., Schulze R. (2009). Intraocular pressure reduction after phacoemulsification with intraocular lens implantation in glaucomatous and nonglaucomatous eyes. Evaluation of a causal relationship between the natural lens and open-angle glaucoma. *Journal of Cataract and Refractive Surgery*.

[5] Shrivastava A., Singh K. (2010). The effect of cataract extraction on intraocular pressure. *Current Opinion in Ophthalmology*.

[6] Fea A. M. (2010). Phacoemulsification versus phacoemulsification with micro-bypass stent implantation in primary open-angle glaucoma. *Journal of Cataract and Refractive Surgery*.

[7] Samuelson T. W., Katz L. J., Wells J. M., Duh Y.-J., Giamporcaro J. E. (2011). Randomized evaluation of the trabecular micro-bypass stent with phacoemulsification in patients with glaucoma and cataract. *Ophthalmology*.

[8] Spiegel D., Garcia-Feijoo J., Martinez de la Casa M. iStent trabecular micro-bypass and concurrent cataract surgery: 24 month results.

[9] Craven E. R. Prospective randomized controlled trial of cataract surgery with trabecular micro-bypass stent in mild-moderate open angle glaucoma: safety in two-year follow-up.

[10] Arriola-Villalòbos P., Martinez-de-la-Casa J. M., Dìaz-Valle D., Fernàndez-Pèrez C., Garcia-Sanchez J., Garcia-Feijoò J. (2012). Combined iStent trabecular micro-bypass stent implantation and phacoemulsification for coexistent open-angle glaucoma and cataract: a long-term study. *British Journal of Ophthalmology*.

[11] Noecker R. J., Herrygers L. A., Anwaruddin R. (2004). Corneal and conjunctival changes caused by commonly used glaucoma medications. *Cornea*.

[12] Broadway D. C., Grierson I., O'Brien C., Hitchings R. A. (1994). Adverse effects of topical antiglaucoma medication. The outcome of filtration surgery. *Archives of Ophthalmology*.

